# Progress of Medical Science in Germany

**Published:** 1875-01-01

**Authors:** E. J. Doering


### PROGRESS OF MEDICAL SCIENCE IN GERMANY.

BY EDMUND J. DOERING, M.D.

I. A CASE OF MEMBRANOUS DYSMENORRHŒA, BY DR. UNSCHULD (*Berl. Klin. Wochenschrift*, NO. 45). II. ON THE USE OF ERGOT IN PNEUMONIA, BY DR. WYCISK (*Allg. Med. Central Zeitung*, NO. 88).

I.

MEMBRANOUS Dysmenorrhœa is certainly one of the most painful affections the physician is called upon to treat. By its regular return, combined with the excruciating pain it produces, this disease may undermine the strongest nervous system and ruin the health of those who thus suffer. But worst of all, is the fact that uncertain as we are as to its etiology, so unsuccessful are we generally in the treatment of this disorder. The successful termination of this variety of dysmenorrhœa in a patient under our care and the rarity of this disease have induced us to publish the following case:

The patient, a lady, twenty-six years of age, unmarried, came to us for treatment in 1872. She is of slender stature, a blonde. First menstruated at the age of seventeen years. Since that time her menses have been accompanied with considerable pain. For the last six years she has noticed the discharge of shreds of mucous membrane at each menstrual period, the latter becoming more and more painful. Bashfulness prevented her from consulting a physician at the time, until finally, her general health failing rapidly, her parents called in a gynæcologist who dilated the cervix after Sims's method, but without producing any amelioration of the symptoms. When she came to Neuenahr, (a famous watering place in Germany,) to consult us, she had entirely despaired of ever regaining her health — emaciated, exhausted, her general health shattered, she presented but little prospects for a cure by our treatment with baths. We, therefore, persuaded the patient to submit also to local treatment. By touch the uterus was found to be retroverted, painful on contact and somewhat enlarged. Slight fluor albus for a short time after each menstrual period. The other organs healthy. As the patient expected her menses in a few days, we ordered only warm mineral baths (97° F.) and douches of the same temperature. The menses were ushered in by distinct febrile symptoms, as great lassitude, chilliness, fever and general constitutional disturbance. These symptoms lasted for about one day and were followed by the menses which appeared scanty, accompanied with severe pain in the back, pelvic pains and headache.

On the third day violent expulsive uterine contractions occurred, so painful as to cause several attacks of syncope. Skin hot and dry, great thirst and quickened pulse. Soon after the shreds of mucous membrane were expelled the pain diminished, but the inflammatory symptoms did not subside until the fifth day, while the flow of blood lasted a few days longer. The patient stated that this series of symptoms occurred regularly every month. Under the microscope the membranes presented the usual appearance as described by Hausmann and other observers.

Having found that the warm baths and douches only seemed to aggravate the symptoms, we concluded to adopt a more strictly antiphlogistic treatment to diminish the hyperæsthesia of the uterus. For that purpose we reduced the temperature of the baths to 80° F., directing the patient to remain for fifteen minutes at a time in each bath. While in the bath a speculum was introduced, made of silver wire and so arranged that the branches at the mouth of the instrument stand twice as far apart as at the end, allowing the water to flow in freely. The baths were always followed by the cold douche. During the intermenstrual period local abstractions of blood were made by means of Braun's hysterotome, followed immediately by a warm bath. We also made a trial with injections of a solution of nitrate of silver into the uterine cavity, but without producing any good results. At the next menstrual period we directed the patient to take twice daily a warm mineral bath (100° F.), omitting the speculum. This plan of treatment was pursued for three months with very satisfactory results, for her general condition improved, the pain greatly diminished, and the discharge of the shreds of mucous membrane did not occur for several consecutive periods, whereas they had formerly appeared regularly every month.

In the spring of 1873 she got worse, and in the summer of the same year, she came again to us for treatment. The same course of treatment was adopted as the year previous, but improved in so far as we directed vaginal injections of cold water (60° F.,) to be made for fifteen minutes twice daily, by means of Kirsch's vaginal irrigator. The condition of the patient again improved rapidly, so that she was able to return home much sooner than the year previous, with renewed energy of life. According to last accounts she enjoys good health, the membranes having appeared but once since August, 1873.

The success of the treatment we attribute mainly to the baths, which contained $CO_2$. These baths increased the local nutrition and improved the tone of the capillaries and uterine muscular fibres, enabling the contents of the uterus to be expelled easier, thus diminishing the pain. Furthermore, the warm baths, the vaginal injections and the local abstractions of blood served to diminish the chronic inflammation. And finally the general condition of the patient improved by the internal use of our mineral springs, the residence in the country and the quiet life in our watering place.

This case proves that Sims's assertion, that the treatment of membranous dysmenorrhœa is only then of use, if directed against the fundamental trouble (usually displacement of the uterus) is incorrect, for in our patient the retroversion of the uterus very probably originated during the course of the disease in consequence of the dysmenorrhœa. And further, by the deficiency of our knowledge as regards the etiology of this disease, only an expectant plan of treatment can be admissible. In conclusion, we also wish to call attention to the falsity of Hausmann's theory, who states that this disease is due, in all cases, to conception: in short, is nothing else but a repeated abortion. Our patient was, for months, under our close and personal observation, and we can assert, positively, that she did not have sexual intercourse with any person; that consequently conception was not the cause of the disease in her case.

II.

It is reasonable to expect that ergot should produce contraction of the blood-vessels just as easy, if not more so, in the lax pulmonary tissue than in the more resistant tissue of other organs, and that consequently anæmia of the lung will take place the same as anæmia of other mucous membranes after the use of ergot. Starting on this hypothesis the author tried this remedy in six cases of croupous pneumonia, and the result demonstrated the correctness of his views. The contraction of the small pulmonary vessels corresponds to a thickening of their walls with a narrowing of the pores, thus diminishing and even stopping further transudation. Finally the anæmia which is produced, by increasing the activity of the lymphatics also promotes absorption and resolution of the inflammation. The following case is a good illustration of the action of ergot in pneumonia: A robust, strong man, fifty years of age, who had been bled on account of the severity of the initial symptoms of an attack of croupous pneumonia, suddenly expectorated great quantities of a serous mucus (nearly two pints during one forenoon) which was found to be strongly albuminous. Alcohol and benzoic acid were given freely but without producing any beneficial effect. Then ergot was administered in ten grain doses every fifteen minutes until five doses had been taken, with the result that the expectoration ceased in two hours, the numerous rales all over the chest disappeared, leaving only some crepitant rale at the seat of the disease, the upper lobe of the right lung. Several relapses were treated likewise with the same prompt effect. In the other cases of pneumonia the remedy was also given with excellent results.

Ergot is contraindicated in emphysema, morbid friability of the cerebral vessels, further, if the exudation is excessive, and finally in patients who are too much exhausted. In a case of emphysema with habitual excessive expectoration ergot was given, causing a serious degree of cyanosis. In another patient with spermatorrhœa and dilatation of the spermatic and scrotal vessels, hypodermic injections of ergot into the scrotum improved the local trouble, but caused a slight attack of apoplexy. We must mention though, that in the last case strychina was also given, internally, which, according to recent investigations, likewise increases the vascular pressure.

